# Letter from A. F. Barnard, M. D.

**Published:** 1890-03

**Authors:** A. F. Barnard

**Affiliations:** St. Mary’s, Ga.


ST. MARY'S, GA., January 13, 1890.

*Managing Editor Atlanta Medical Journal, Atlanta, Ga.:*

DEAR SIR—In the May number of the *Medical Brief* (a journal published in St. Louis, Mo.), for the year 1889, appears an article from the pen of Dr. W. F. Ellord, of Banner, Mississippi, in which he states, "that on May 1st he delivered a woman of a child which had three coils of the umbilical cord around its neck. The child was well matured but dead; and had been for twenty-four hours, according to the mother's statement." The above case may or may not be remarkably unique; however I desire to place before the readers of your valuable journal a case similar in one important feature, but essentially different in others, as the sequel will show. For whereas in Dr. E's case there were three coils of cord around the child's neck, in the one I now present there were not only three coils around the neck, but one around the body as well, the cord being unusually long and attenuated. And while in this former case death occurred *in utero*, in the latter the child is well and hearty at this time, although at its birth death seemed imminent from asphyxiation.

The mother is now about forty-four years of age, and has two boys, aged fifteen and thirteen respectively. She has not been pregnant to my knowledge during the intervening years.

By a strange coincidence the two cases herein specified occurred in the same year and in the same month, viz.: May, 1889.

Very respectfully,

A. F. BARNARD.

